# Toward Industrial Application of Cyanobacterial Biosorption: Insights From Real Electroplating Effluents

**DOI:** 10.1002/wer.70366

**Published:** 2026-03-31

**Authors:** Matilde Ciani, Chiara Capelli, Giulia Daly, Roberto de Philippis, Alessandra Adessi

**Affiliations:** ^1^ Department of Agriculture, Food, Environment and Forestry (DAGRI) University of Florence Florence Italy

## Abstract

Galvanic electrodeposition is widely used in surface engineering, making the sustainable management of metal‐loaded effluents a future challenge. Although cyanobacteria‐based biosorption has been recognized as an eco‐friendly strategy to remove metals, its application to real wastewater has not been explored. This study investigates cyanobacterial potential in the treatment of different electroplating wastewater originated from Cu, Ni, Au, and Pd‐plating baths. The results revealed that pH, metal concentration, and composition of the plating bath influenced biosorption. Notably, Ni‐specific uptake reached 3 mmol of Ni per gram of biomass dry weight, exceeding values reported in the literature. In contrast, for precious metals, the uptake was lower than 0.1 mmol per gram of biomass dry weight. This study represents a first step in the scaling‐up of this cyanobacteria‐based biotechnology, highlighting that higher biomass concentration and combined approaches are needed to improve removal efficiency while producing clean water.

## Summary

- Cyanobacteria effectively remove nickel from real electroplating effluents, with specific uptake reaching 2.9 mmol per gram dry weight.
- Precious metal uptake is lower than in synthetic solutions due to high ionic strength in industrial plating baths.
- Integrating cyanobacterial biosorption with conventional methods may promote metal removal and valorization from complex industrial electroplating effluents.

## Graphical abstract

The study demonstrates that exopolysaccharide‐producing cyanobacteria are effective biosorbents for metals contained in electroplating wastewater. Ni uptake (2.3–2.9 mmol/g) was higher than for precious metals such as palladium (Pd) and gold (Au) (lower than 0.1 mmol/g). *D. salina* 16Som2 and *Cyanothece* sp. CE 4 showed the best performances.

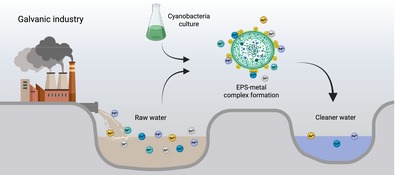

## 1 Introduction

Galvanic electrodeposition is a key technology in surface engineering, enhancing material appearance, functionality, and durability across sectors through the deposition of metallic ions from aqueous solutions to the surface of materials (Giurlani et al. 2018). Nevertheless, the environmental impact of the sector is substantial: Around 29% of the waste streams generated by this industry are classified as toxic or hazardous, containing cyanide (CN^−^) and heavy metals such as copper (Cu), zinc (Zn), arsenic (As), beryllium (Be), cadmium (Cd), lead (Pb), hexavalent chromium (Cr(VI)), and nickel (Ni). Heavy metals are non‐degradable and can accumulate in the environment, causing harmful effects on human health and ecosystems if not properly treated and discharged (Kamar et al. 2022; Rajoria et al. 2022; Ramírez Calderón et al. 2020). Also, many electrodepositions use precious metals, like gold (Au), palladium (Pd), and platinum (Pt), making their recovery from effluents desirable for economic and circular economy reasons (Adams et al. 2024). Despite strict regulations, high energy consumption, and costs (Giurlani et al. 2018), the electroplating market size, valued at USD 21.7 billion in 2025, is projected to exceed USD 32.4 billion by 2033, with a CAGR (compound annual growth rate) of 4.1% (Electroplating Market 2023). Thus, the next challenge for the future of this industry is represented by the sustainable management of effluents and chemicals at low investment costs, including treatment and recovery processes (Giurlani et al. 2018; Kamar et al. 2022).

Cyanobacteria have been proposed as promising instruments to remove metal ions from aqueous solutions due to the production of exopolysaccharides (EPS) characterized by anionic charge. This charge is due to the presence of sulfate, phosphate, pyruvate groups, and uronic acids and is responsible for the binding with positively charged metals (Ghorbani 2022; Mota et al. 2022; Olguín et al. 2022). While biological metal removal can occur through both bioaccumulation, an active, metabolism‐dependent process of intracellular metal intake, and biosorption, a passive mechanism involving the binding of ions to the cell surface, this study focuses specifically on biosorption (Parmar and Patel 2025). Biosorption is mostly driven by the physicochemical interactions between metal ions and the anionic functional groups present in cyanobacterial EPS (Ciani et al. 2024). Given that these interactions are rapid and do not require metabolic activity, biosorption may offer a more robust and cost‐effective pathway for treating industrial effluents where high metal toxicity might otherwise inhibit living cell metabolism. Additionally, cyanobacteria‐based biosorption presents other advantages: the high surface‐to‐volume ratio and EPS productivity, the interesting physicochemical properties, the possible exploitation of wastewaters as nutrient sources and of the biomass for multiple biosorption cycles (Mota et al. 2016; Ordóñez et al. 2023). Moreover, there are still unexplored opportunities in the valorization of the biomass obtained after biosorption. Indeed, new metallic‐organic materials can be generated through the biosorption process and exploited, for example, in the catalysis and nanotechnology sectors, or as antimicrobials (Ciani and Adessi 2023). Although many studies have evaluated the metal biosorption potential of cyanobacteria adopting laboratory‐prepared metallic solutions, there are just a few examples of applications with real wastewater (Ordóñez et al. 2023). To our knowledge, even if the research studies related to metal biosorption by cyanobacteria are increasing over time, only a limited number of published studies (Colica et al. 2010; Zinicovscaia et al. 2019) have previously investigated biosorption and bioaccumulation from real electroplating effluents using a cyanobacterium. Crucially, it remains unknown if the biosorption process studied adopting a synthetic solution is representative of the process carried out in a complex chemical matrix of authentic industrial effluents, characterized by high ionic strength and the presence of specific competing ions, leaving a significant gap in the understanding of their performance in real‐world systems and industrial applicability. Given that the biosorption process is affected by metal concentration, pH, and also by the presence of coexisting ions in the solutions, more research is needed to confirm its industrial applicability. Building on a previous work, in which EPS‐producing cyanobacterial strains were tested in mono‐ and multi‐metallic synthetic solutions by characterization of biosorption mechanisms through kinetics and adsorption isotherms (Ciani et al. 2024), here their application was extended to real electroplating effluents. Thus, this study aimed to bridge the gap between laboratory‐scale research on synthetic systems and the complex conditions of industrial wastewater. Testing cyanobacterial biosorption in such realistic matrices provides essential insights into both opportunities and limitations, thereby paving the way for process optimization and future integration into industrial wastewater treatment and circular economy strategies.

## 2 Experimental

### 2.1 Cyanobacteria and Electroplating Effluents

Two unicellular cyanobacteria, namely, *Cyanothece* sp. CE 4 (Genbank: OQ945752) and *Dactylococcopsis salina* sp. 16Som2 (Genbank: OQ945751), and a co‐culture of *Cyanothece*‐like cells named Vi22 M were cultivated using a seawater‐enriched medium as previously described (Ciani et al. 2024). The cultures were maintained under continuous illumination (200‐μmol photons m^−2^ s^−1^) by a white LED lamp and insufflation of CO_2_‐enriched air (99:1, v:v) at a flow rate of 0.2 L per L of culture per minute, at a constant temperature of 26°C ± 0.5°C and pH 8.5 ± 0.5. The strains were selected for their ability to produce EPS and remove Cu, Ni, and Zn from single and multi‐metal solutions (Ciani et al. 2024).

Five effluents from Cu, Ni, Pd, or Au electroplating baths were provided by Galvanica Aricci Srl (Ghisalba, BG, Italy). The concentration of the main metals was quantified by ICP‐OES (iCAP 7400 ICP‐OES Analyzer, Thermo Fisher Scientific, USA) following the method APAT CNR IRSA 3020 Man 29 2003; the pH was measured with a pH meter (pH 50, XS Instruments, Italy).

### 2.2 Biosorption Experiments

**FIGURE 1:**
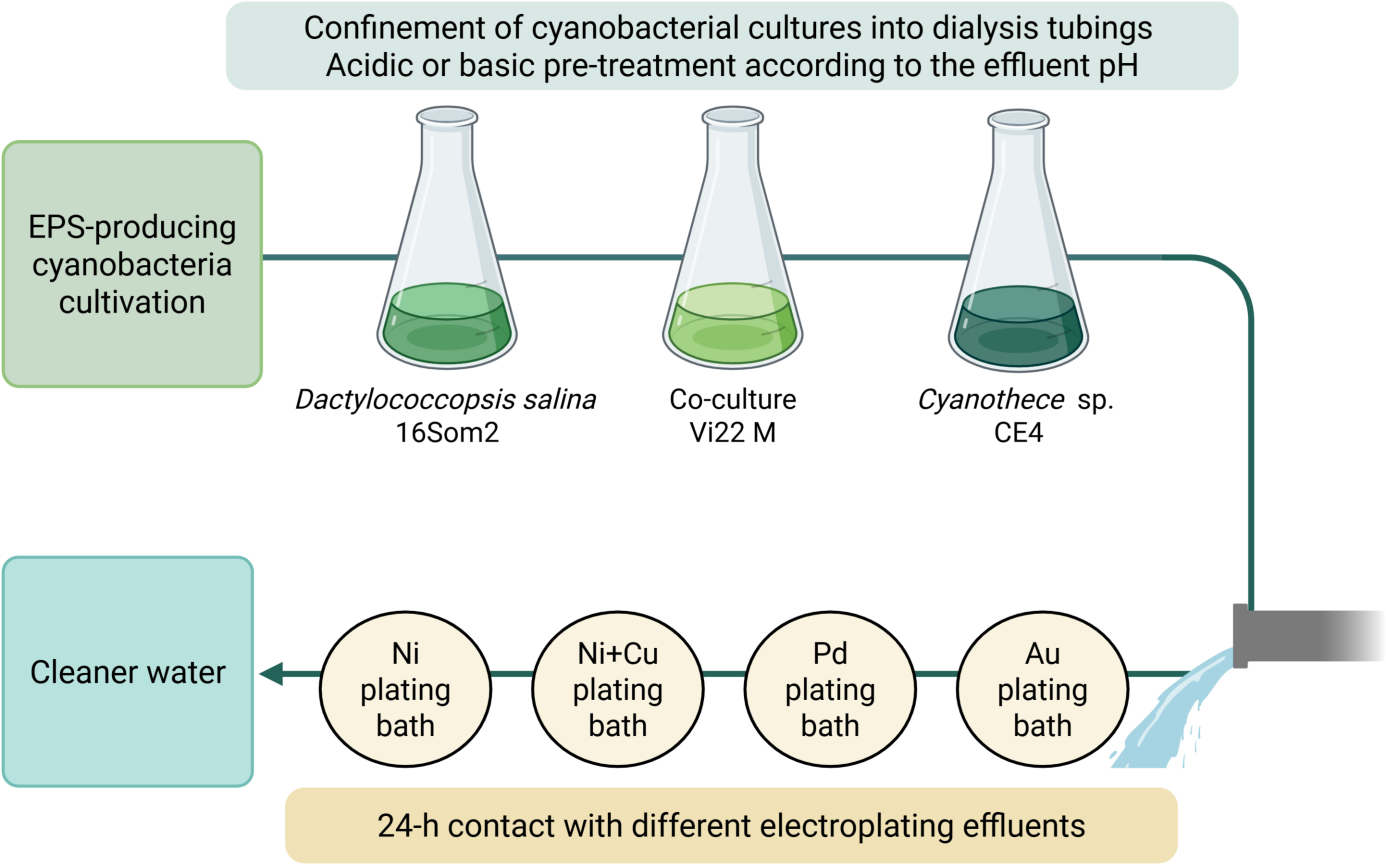
Main workflow of the experiment.

One week‐grown cultures were harvested, confined in dialysis tubing (MW cut‐off 12–14 kDa, S/V 1.9 cm^−1^, Medicell Membranes Ltd., UK) and pre‐treated with HCl or NaOH 0.1 M, as previously described in Ciani et al. (2024). Acidic or alkaline pre‐treatment was selected depending on the pH of the effluents to avoid altering metal solubility. The same pre‐treatments were carried out to the cultivation medium without biomass, considered as abiotic control to account for abiotic metal losses. The biomass dry weight (DW, g L^−1^) of pre‐treated cultures was determined by filtering cell suspension on pre‐weighted 0.45‐μm filters and then dried at 105°C for 4 h. Pre‐treated cultures and the abiotic controls were confined in small dialysis tubings (MW cut‐off 12–14 kDa, S/V 3.7 cm^−1^, Medicell Membranes Ltd., UK), dipped in 100‐mL glass cylinders containing the effluents with a ratio 1:10 (v/v, biosorbent/effluent) under continuous stirring (200 rpm) at 25°C. Three replicates were set for each condition. After 24 h, residual metal concentration in the effluents was determined by ICP‐OES as described above and adopted to calculate the specific metal uptake expressed as moles of metal removed by the biomass (compared to the abiotic control) per gram of biomass dry weight (mmol or μmol g^−1^ DW). The experimental flow is summarized in Figure 1.

### 2.3 Data Analysis

Results are expressed as the mean of three replicates ± standard deviation (SD). Statistical significance of biosorbent, effluent type, and their interaction was assessed using two‐way ANOVA and Tukey's test (*p* < 0.05) with OriginLab Pro Software.

## 3 Results and Discussion

### 3.1 Effluent Characterization and Biomass Pre‐Treatment

The electroplating effluents exhibited significant variability in metal composition (from a few to hundreds of mg L^−1^ of the specific metal characterizing the plating bath, with some effluents notably rich in Ni) and pH, ranging from highly acidic (pH 1.66) to near‐neutral (pH 7.19) (Table 1). Due to the extreme acidity and high chemical stability of the original Cu plating bath, initial biosorption attempts were unsuccessful, and chemical neutralization was found ineffective. Also, adjusting the pH of industrial effluents is often expensive and can trigger the precipitation of metals. Furthermore, in effluents containing cyanide, alkalization poses significant safety risks due to the potential liberation of toxic fumes (Rudnicki et al. 2014). To overcome these limitations and simulate industrial equalization processes (Ika Pratiwi et al. 2021), the Cu bath was integrated to the Ni(1) bath at a 4:1 (Ni(1):Cu; v:v) ratio, aiming to reduce pH and Ni concentration of Ni(1) bath (Table 1) while achieving self‐neutralization of Cu bath. The mixed Ni(1) + Cu effluent was characterized by a pH of 2.41 and Ni and Cu concentrations of 598 and 6 mg L^−1^, respectively.

**TABLE 1:** pH and main metals' composition of five effluents provided by Galvanica Aricci Srl.

Metal plating bath	pH	Au	Cr	Cu	Ni	Pd	Zn
		mg L^−1^					
Cu^a^	1.66	< 0.01	0.01	29.52	0.29	nd^b^	21.19
Ni(1)^a^	6.67	nd	nd	0.19	747.22	nd	0.75
Ni(2)	4.11	nd	nd	0.67	386.94	nd	1.01
Pd	7.19	< 0.01	0.16	7.31	0.64	24.55	0.14
Au	5.30	7.22	4.49	0.25	6.30	nd	1.66

^a^ Cu and Ni(1) plating baths were mixed to obtain the Ni(1) + Cu effluent.

^b^ nd, not detected.

It is known that at pH below 2 or above 6, the removal of metals is reduced for the ionization state of functional groups, the speciation and solubility of metal ions (Godlewska‐Żyłkiewicz et al. 2019; Priyadarshanee and Das 2021; Shen and Chirwa 2019). Thus, acidic or basic pre‐treatment of the biomass was selected according to the pH of the effluent (Table 1). It is known that pre‐treatment influences metal biosorption by either increasing binding capacity through the removal of competing cations or, in some cases, negatively impacting it by altering the biomass surface (Martínez‐Macias et al. 2024; Murty et al. 2026). These processes specifically target and modify functional groups such as carboxyl, amino, hydroxyl, and amides, which serve as the primary binding sites. Furthermore, while HCl‐pretreatment cleans and frees these sites, alkali (NaOH) treatment promotes deprotonation of the functional groups; this can cause an increase in the solution's pH during the biosorption process, which may affect metal solubility and risk the precipitation of metals as insoluble hydroxides if the pH rises above 6 (Kumar and Gaur 2011; Martínez‐Macias et al. 2024; Murty et al. 2026). Consequently, HCl or NaOH pre‐treated biosorbents (characterized by pH 3.5 ± 0.3 or 10.1 ± 0.2, respectively) were adopted for Ni(1) + Cu/Ni(2) or Pd/Au plating baths, respectively. The dry weight of pre‐treated cultures after 1 week of growth was 0.6, 1.0, and 0.8 g L^−1^, for 16Som2, Vi22 M, and CE 4, respectively. At the end of the biosorption experiment, the pH was 2.58, 6.06, 5.80, and 4.18, respectively.

### 3.2 Ni Effluents

**FIGURE 2:**
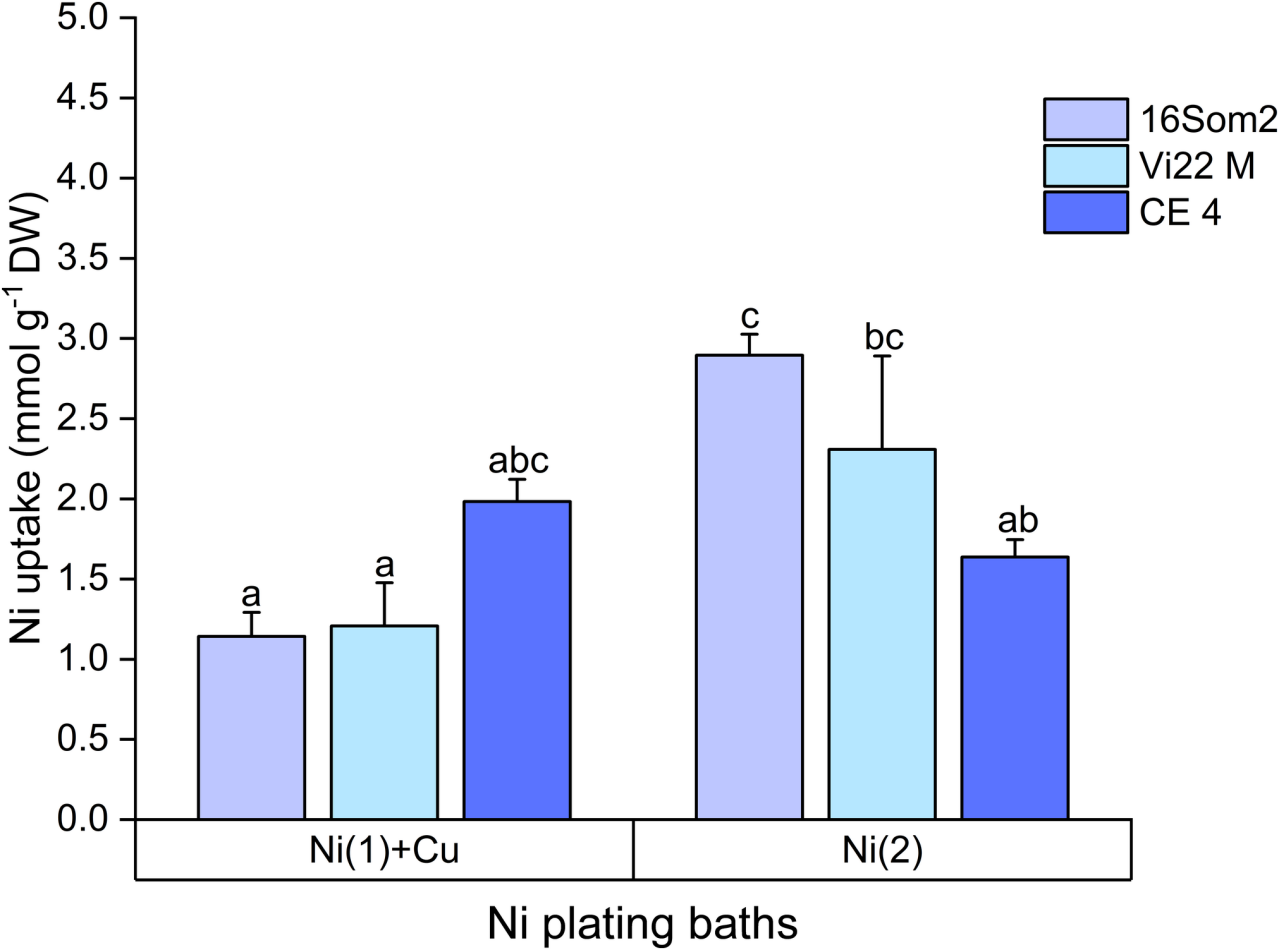
Ni specific uptake (mmol Ni g^−1^ DW) from Ni(1) + Cu and Ni(2) plating baths after 24 h of contact with 16Som2, Vi22 M, and CE 4. Different letters mean statistically significant differences between groups (*p* < 0.05).

Given their high Ni content, Ni uptake from Ni(1) + Cu and Ni(2) effluents was compared in Figure 2, while the uptake of Cu and Zn was always lower than 20 μmol g^−1^ DW (Tables S1 and S2). Two‐way ANOVA revealed that both the effluent type and its interaction with biosorbents significantly affected Ni uptake (*p* < 0.005), suggesting that pH and effluent composition impacted biosorption efficiency. The Ni uptake capacity observed in this study (up to 2.9 mmol g^−1^ DW by 16Som2 in Ni(2) bath) is highly noteworthy within the range of reported values for cyanobacterial biosorbents (Ciani et al. 2024; Zinicovscaia et al. 2019). While direct comparisons are limited due to differences in experimental conditions, such as biomass dosage, contact time, and pH, these findings emphasize that the performance was maintained despite the complex matrix and the presence of competing ions, which typically reduce the availability of binding sites compared to synthetic solutions. The higher molar concentration of Ni (10.2–6.6 mmol L^−1^) likely triggered an antagonistic interaction between different metallic ions, where Ni ions saturated the majority of the available binding sites of cyanobacteria‐based biosorbents. This competitive inhibition, combined with the high chemical stability of Cu in the original plating bath, may explain why no significant Cu removal was observed from Ni(1) + Cu effluent.

Comparisons with studies on artificial solutions and other biosorbents underscored the efficiency of cyanobacteria for real effluents (Ciani et al. 2024; Sundararaju et al. 2022). The observed high uptake is consistent with the trend previously indicated by the isotherm studies conducted on artificial solutions (Ciani et al. 2024). While the Langmuir and Freundlich models poorly explained Zn uptake, the models consistently showed a strong fit for Cu and Ni across most cyanobacterial strains tested, suggesting well‐defined interaction mechanisms. Comparison with fungal‐based biosorption (Sundararaju et al. 2022) emphasized cyanobacteria's efficiency at lower biomass concentrations, highlighting the trade‐off between biosorbent dosage and removal efficiency as suggested by a recent study carried out with the same cyanobacteria (Ciani et al. 2024).

Previous studies utilizing membranes specifically designed for acidic conditions reported minimal sorption efficiencies for synthetic Cu(II), Ni(II), and Zn(II) solutions at pH values between 1.0 and 2.0 (Rudnicki et al. 2014). This low performance was attributed to intense proton competition for binding sites, a challenge further compounded by high ionic strength (e.g., NaCl concentrations), which covered functional groups and limited ion exchange (Rudnicki et al. 2014). The mixing of the two effluents (Ni(1) and Cu) to improve biosorption conditions of Cu resulted in no improvement, despite the alkaline pre‐treatment of the cyanobacteria‐based biosorbents, which was expected to enhance metal uptake from acidic effluents (Blais et al. 2003). To address such extreme conditions (i.e., pH and stability) in future applications, cyanobacteria could be subjected to Adaptive Laboratory Evolution (ALE) to select for acid‐tolerant phenotypes (Liu et al. 2019), or genetically engineered to overexpress specific EPS components to maximize metal biosorption (Al‐Amin et al. 2021).

### 3.3 Pd and Au Effluents

**FIGURE 3:**
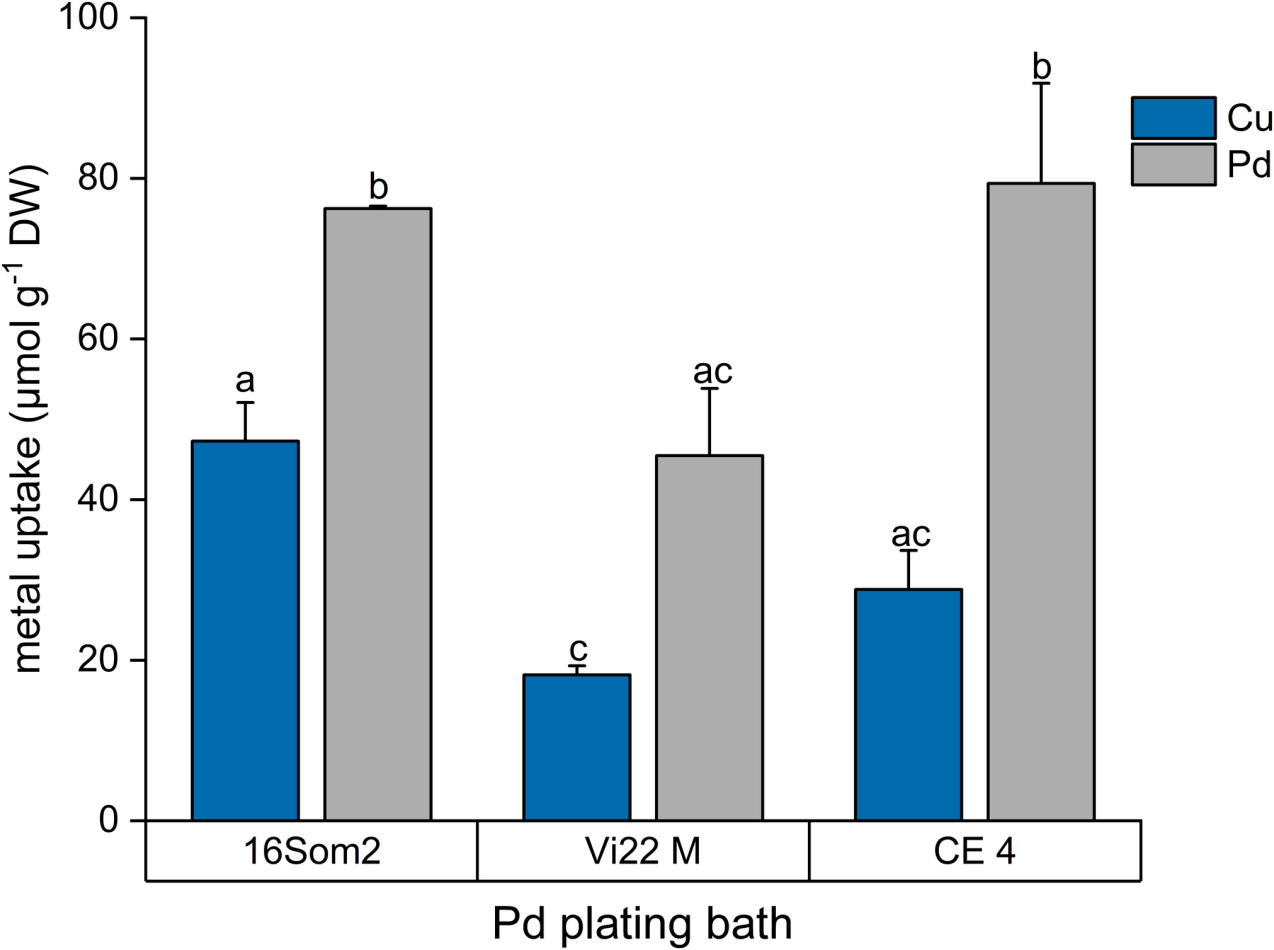
Metal specific uptake (mmol g^−1^ DW) from Pd plating bath after 24 h of contact with 16Som2, Vi22 M, and CE 4. Different letters mean statistically significant differences between groups (*p* < 0.05).

Pd and Au plating baths appeared suitable for cyanobacteria‐based biosorption due to their lower metal concentrations and held particular interest for recovering precious metals (Tiwari et al. 2022). However, specific uptake of single metal was generally below 0.1 mmol/g DW (Tables S3 and S4), yet significant uptake differences were observed among strains and metals. Two‐way ANOVA confirmed that biosorbent type and metal identity significantly influenced uptake (*p* < 0.05). 16Som2 showed a significantly higher Cu and Pd uptake from Pd plating bath compared to Vi22M (*p* < 0.05 and 0.0001, respectively, Figure 3), reaching 0.12‐mmol Cu + Pd g^−1^ DW. These findings are consistent with those observed in Ni‐rich effluents, highlighting the role of initial metal concentration on metal uptake. Indeed, the initial molar concentration of Pd (0.23 mmol L^−1^) was higher than that of the other species present (Cu 0.12 mmol L^−1^; Cr, Ni, Zn ≤ 0.01 mmol L^−1^). This molar dominance likely triggered an antagonistic interaction between different metallic ions. While variables such as pH, temperature, and biomass pre‐treatment are known to modulate biosorption capacity from multimetallic solutions (Sieber et al. 2024), these results suggest that the concentration gradient remains a primary determinant of metal preference in complex industrial matrices. Generally, CE 4 revealed the highest Pd uptake (79.4 ± 12.5 μmol g^−1^ DW), followed by 16Som2 (76.2 ± 0.3 μmol g^−1^ DW).

**FIGURE 4:**
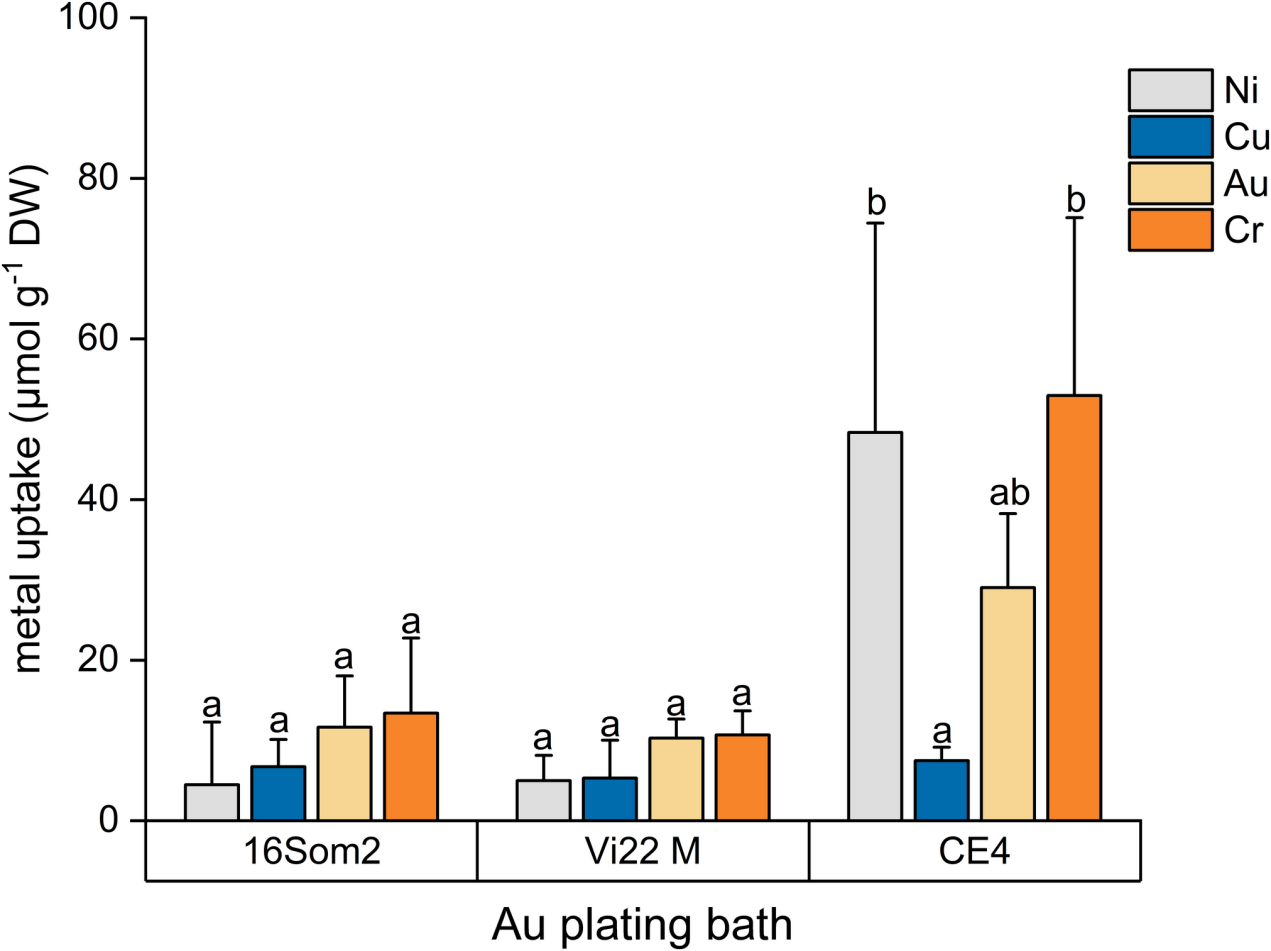
Metal specific uptake (mmol g^−1^ DW) from Au plating bath after 24 h of contact with 16Som2, Vi22 M, and CE 4. Different letters mean statistically significant differences between groups (*p* < 0.05).

CE 4 demonstrated also superior Au, Ni, and Cr uptake from Au plating bath (29.0 ± 9, 48.4 ± 26.1, and 53.0 ± 22.2 μmol g^−1^ DW, respectively), showing fourfold higher total metal uptake (0.14 mmol g^−1^ DW, Table S4) than the other cultures (Figure 4).

Cu uptake values were comparable to those observed by Zinicovscaia et al. from other real electroplating wastewaters adopting *A. platensis* (Zinicovscaia et al. 2019). In contrast, Shen and Chirwa (Shen and Chirwa 2019) used the microalga *T. obliquus* to remove Au from a lab‐prepared solution, and the uptake reached 50 mg Au g^−1^ DW (corresponding to 0.25 mmol Au g^−1^ DW), nearly twice the values reported in this study, likely reflecting the lack of competitive ions and complexing agents present in real industrial matrices. Indeed, plating baths containing precious metals, like Au and Pd, are rich in conductive salts to reduce the resistance of the solutions (Galvanica Aricci Srl, personal communication). It is known that the presence of coexisting ions can negatively affect the biosorption process (Aranda‐García et al. 2020), probably reducing metal uptake from Pd and Au plating baths compared to Ni plating baths. Since the removal of metals from wastewater at concentrations lower than 10 mg L^−1^ is often difficult with conventional techniques (Kamar et al. 2022) while cyanobacteria are more effective at low metal concentrations (Ciani and Adessi 2023), the selection of the appropriate effluent or the combination of cyanobacteria‐based biosorption following other physico‐chemical methods may represent a valid strategy to maximize the removal, overcoming the low removal efficiency here observed. Also, rather than viewing biosorption as a standalone bioremediation step, industrial scalability can be reached by utilizing EPS‐producing cyanobacteria as bio‐concentrators, effectively transforming low‐concentration effluents into metal‐saturated organic precursors that bridge the gap between waste treatment and secondary raw material production. As an example, Cu‐ and Zn‐enriched biomass of *D. salina* 16Som2, obtained after biosorption from synthetic metallic solutions, has already shown promising results for valorization as a hybrid catalyst for pharmaceutical reactions (Ciani et al. 2026). While the detailed structural characterization (e.g., SEM–EDX, FTIR, and XAS) of the metal–organic biomass has already been established in that study, the current study has focused on validating the capture efficiency and scalability of the process using authentic industrial effluents. The successful recovery of Ni, Pd, and Au from such complex matrices is a critical first step; however, further analytical studies are required to confirm if the proprietary additives and high ionic strength of these real‐world effluents alter the catalytic properties or the surface morphology of the resulting biomass‐based materials.

## 4 Conclusions

This study provides a critical assessment of metal removal with cyanobacteria by moving from synthetic laboratory solutions to real industrial electroplating effluents. The results demonstrate that while the strains achieved high specific Ni uptake capacities (up to 3 mmol g^−1^), the overall removal efficiency was constrained by the high initial metal loads and biosorbent dosage. This necessitates a strategic optimization of the biosorbent‐to‐sorbate ratio: while lower dosages maximize specific uptake for metal recovery and valorization, higher dosages or multi‐stage configurations are required to meet stringent discharge targets.

Furthermore, the lower uptake observed for precious metals (Au and Pd) compared to Ni suggests that the complex chemical matrix of real effluents, characterized by high ionic strength and competing ions, can also contribute to a reduction in binding site availability. The presence of stable metal complexes and high buffering capacities in these effluents identifies the gap between theoretical laboratory capacity and industrial performance. Consequently, future research must shift toward multi‐stage contact systems by adopting combined removal techniques and biomass immobilization to enhance selectivity and achieve total removal percentages compatible with industrial wastewater standards.

## Author Contributions

**Matilde Ciani:** conceptualization, methodology, formal analysis, data curation, writing – original draft, investigation. **Chiara Capelli:** formal analysis, data curation, writing – review and editing. **Giulia Daly:** writing – review and editing, data curation. **Roberto de Philippis:** conceptualization, methodology, supervision, funding acquisition, writing – review and editing. **Alessandra Adessi:** conceptualization, supervision, supervision, funding acquisition, writing – review and editing.

## Conflicts of Interest

The authors declare no conflicts of interest.

## Supporting information


**Table S1:** Specific uptake from Ni(1) + Cu effluent.
**Table S2:** Specific uptake from Ni(2) effluent.
**Table S3:** Specific uptake from Pd effluent.
**Table S4:** Specific uptake from Au effluent.

## Data Availability

The data supporting this article have been included as part of the Supporting Information.
