# Supplementary material for: Toward Industrial Application of Cyanobacterial Biosorption: Insights From Real Electroplating Effluents
Source: Water Environ Res. 2026 Mar 31;98(4):e70366. doi: 10.1002/wer.70366 (PMC13037690; doi:10.1002/wer.70366)
Supplement: Supplementary file 1 — Table S1: Specific uptake from Ni(1) + Cu effluent. Table S2: Specific uptake from Ni(2) effluent. Table S3: Specific uptake from Pd effluent. Table S4: Specific uptake from Au effluent. [file WER-98-e70366-s001.docx]

**Towards industrial application of cyanobacterial biosorption: insights from real electroplating effluents**

Matilde Ciani, Chiara Capelli, Giulia Daly, Roberto de Philippis and Alessandra Adessi

*Department of Agriculture, Food, Environment and Forestry (DAGRI), University of Florence, Piazzale delle Cascine, 18, 50144 Florence, Italy*

**Supporting Information**

**Table S1** Specific uptake from Ni(1)+Cu effluent

| **Biosorbent** | | **Specific uptake** | | | | | |
| --- | --- | --- | --- | --- | --- | --- | --- |
| **Culture** | **Concentration** | **Ni** | | **Cu** | | **Zn** | |
|  | **g/L (DW)** | **mg/g** | **mmol/g** | **mg/g** | **mmol/g** | **mg/g** | **mmol/g** |
| 16SOM2 | 0.60 | 59.95 | 1.02 | 0.39 | 0.01 | 0.18 | 0.00 |
|  | 0.60 | 76.90 | 1.31 | 0.83 | 0.01 | 0.45 | 0.01 |
|  | 0.60 | 64.33 | 1.10 | 0.21 | 0.00 | 0.00 | 0.00 |
| VI22 | 1.00 | 65.34 | 1.11 | 0.57 | 0.01 | 0.24 | 0.00 |
|  | 1.00 | 88.77 | 1.51 | 1.02 | 0.02 | 0.64 | 0.01 |
|  | 1.00 | 58.60 | 1.00 | 0.68 | 0.01 | 0.40 | 0.01 |
| CE4 | 0.80 | 110.69 | 1.89 | 1.45 | 0.02 | 0.33 | 0.01 |
|  | 0.80 | 122.22 | 2.08 | 2.25 | 0.04 | 0.62 | 0.01 |
|  | 0.80 | na | na | na | na | na | na |

**Table S2** Specific uptake from Ni(2) effluent

| **Biosorbent** | | **Specific uptake** | | | | | |
| --- | --- | --- | --- | --- | --- | --- | --- |
| **Culture** | **Concentration** | **Ni** | | **Cu** | | **Zn** | |
|  | **g/L (DW)** | **mg/g** | **mmol/g** | **mg/g** | **mmol/g** | **mg/g** | **mmol/g** |
| 16SOM2 | 0.60 | 168.81 | 2.88 | 0.00 | 0.00 | 1.72 | 0.03 |
|  | 0.60 | 162.87 | 2.77 | 0.00 | 0.00 | 0.73 | 0.01 |
|  | 0.60 | 178.24 | 3.04 | 0.00 | 0.00 | 1.42 | 0.02 |
| VI22 | 1.00 | 155.21 | 2.64 | 0.00 | 0.00 | 0.72 | 0.01 |
|  | 1.00 | 155.28 | 2.65 | 0.00 | 0.00 | 0.62 | 0.01 |
|  | 1.00 | 96.01 | 1.64 | 0.00 | 0.00 | 1.02 | 0.02 |
| CE4 | 0.80 | 91.61 | 1.56 | 0.00 | 0.00 | 0.00 | 0.00 |
|  | 0.80 | 100.63 | 1.71 | 0.00 | 0.00 | 0.00 | 0.00 |
|  | 0.80 | 37.78 | 0.64 | 0.00 | 0.00 | 0.00 | 0.00 |

**Table S3** Specific uptake from Pd effluent

| **Biosorbent** | | **Specific uptake** | | | | | | | | | |
| --- | --- | --- | --- | --- | --- | --- | --- | --- | --- | --- | --- |
| **Culture** | **Concentration** | **Ni** | | **Cu** | | **Zn** | | **Cr** | | **Pd** | |
| **Biosorbent** | **g/L (DW)** | **mg/g** | **mmol/g** | **mg/g** | **mmol/g** | **mg/g** | **mmol/g** | **mg/g** | **mmol/g** | **mg/g** | **mmol/g** |
| 16SOM2 | 0.60 | 0.10 | 0.00 | 2.79 | 0.04 | 0.18 | 0.00 | 0.00 | 0.00 | 8.14 | 0.08 |
|  | 0.60 | 0.18 | 0.00 | 3.22 | 0.05 | 0.10 | 0.00 | 0.01 | 0.00 | 8.09 | 0.08 |
|  | 0.60 | 0.00 | 0.00 | 0.00 | 0.00 | 0.24 | 0.00 | 0.00 | 0.00 | 0.00 | 0.00 |
| VI22 | 1.00 | 0.06 | 0.00 | 1.10 | 0.02 | 0.04 | 0.00 | 0.01 | 0.00 | 4.21 | 0.04 |
|  | na | na | na | na | na | na | na | na | na | na | na |
|  | 1.00 | 0.07 | 0.00 | 1.21 | 0.02 | 0.00 | 0.00 | 0.01 | 0.00 | 5.47 | 0.05 |
| CE4 | 0.80 | 0.05 | 0.00 | 2.05 | 0.03 | 0.00 | 0.00 | 0.03 | 0.00 | 8.39 | 0.08 |
|  | 0.80 | 0.10 | 0.00 | 1.48 | 0.02 | 0.00 | 0.00 | 0.00 | 0.00 | 7.15 | 0.07 |
|  | 0.80 | 0.11 | 0.00 | 1.97 | 0.03 | 0.01 | 0.00 | 0.01 | 0.00 | 9.80 | 0.09 |

**Table S4** Specific uptake from Au effluent

| **Biosorbent** | | **Specific uptake** | | | | | | | | | |
| --- | --- | --- | --- | --- | --- | --- | --- | --- | --- | --- | --- |
| **Culture** | **Concentration** | **Ni** | | **Cu** | | **Zn** | | **Au** | | **Cr** | |
|  | **g/L (DW)** | **mg/g** | **mmol/g** | **mg/g** | **mmol/g** | **mg/g** | **mmol/g** | **mg/g** | **mmol/g** | **mg/g** | **mmol/g** |
| 16SOM2 | 0.60 | 0.00 | 0.00 | 0.41 | 0.01 | 0.00 | 0.00 | 1.58 | 0.01 | 0.33 | 0.01 |
|  | 0.60 | 0.79 | 0.01 | 0.65 | 0.01 | 0.00 | 0.00 | 1.56 | 0.01 | 0.51 | 0.01 |
|  | 0.60 | 0.00 | 0.00 | 0.22 | 0.00 | 0.00 | 0.00 | 3.75 | 0.02 | 1.25 | 0.02 |
| VI22 | 1.00 | 0.37 | 0.01 | 0.13 | 0.00 | 0.00 | 0.00 | 1.55 | 0.01 | 0.42 | 0.01 |
|  | 1.00 | 0.08 | 0.00 | 0.20 | 0.00 | 0.00 | 0.00 | 2.04 | 0.01 | 0.73 | 0.01 |
|  | 1.00 | 0.43 | 0.01 | 0.68 | 0.01 | 0.00 | 0.00 | 2.49 | 0.01 | 0.51 | 0.01 |
| CE4 | 0.80 | 1.31 | 0.02 | 0.37 | 0.01 | 0.00 | 0.00 | 3.90 | 0.02 | 1.60 | 0.03 |
|  | 0.80 | 4.37 | 0.07 | 0.58 | 0.01 | 0.00 | 0.00 | 7.53 | 0.04 | 3.91 | 0.08 |
|  | 0.80 | 2.84 | 0.05 | 0.48 | 0.01 | 0.00 | 0.00 | 5.72 | 0.03 | 2.75 | 0.05 |
